# Supplementary material for: Sphingomonas sediminicola Is an Endosymbiotic Bacterium Able to Induce the Formation of Root Nodules in Pea (Pisum sativum L.) and to Enhance Plant Biomass Production
Source: Microorganisms. 2023 Jan 12;11(1):199. doi: 10.3390/microorganisms11010199 (PMC9861922; doi:10.3390/microorganisms11010199)
Supplement: Supplementary file 1 [file microorganisms-11-00199-s001.zip › Figure S1.pdf]

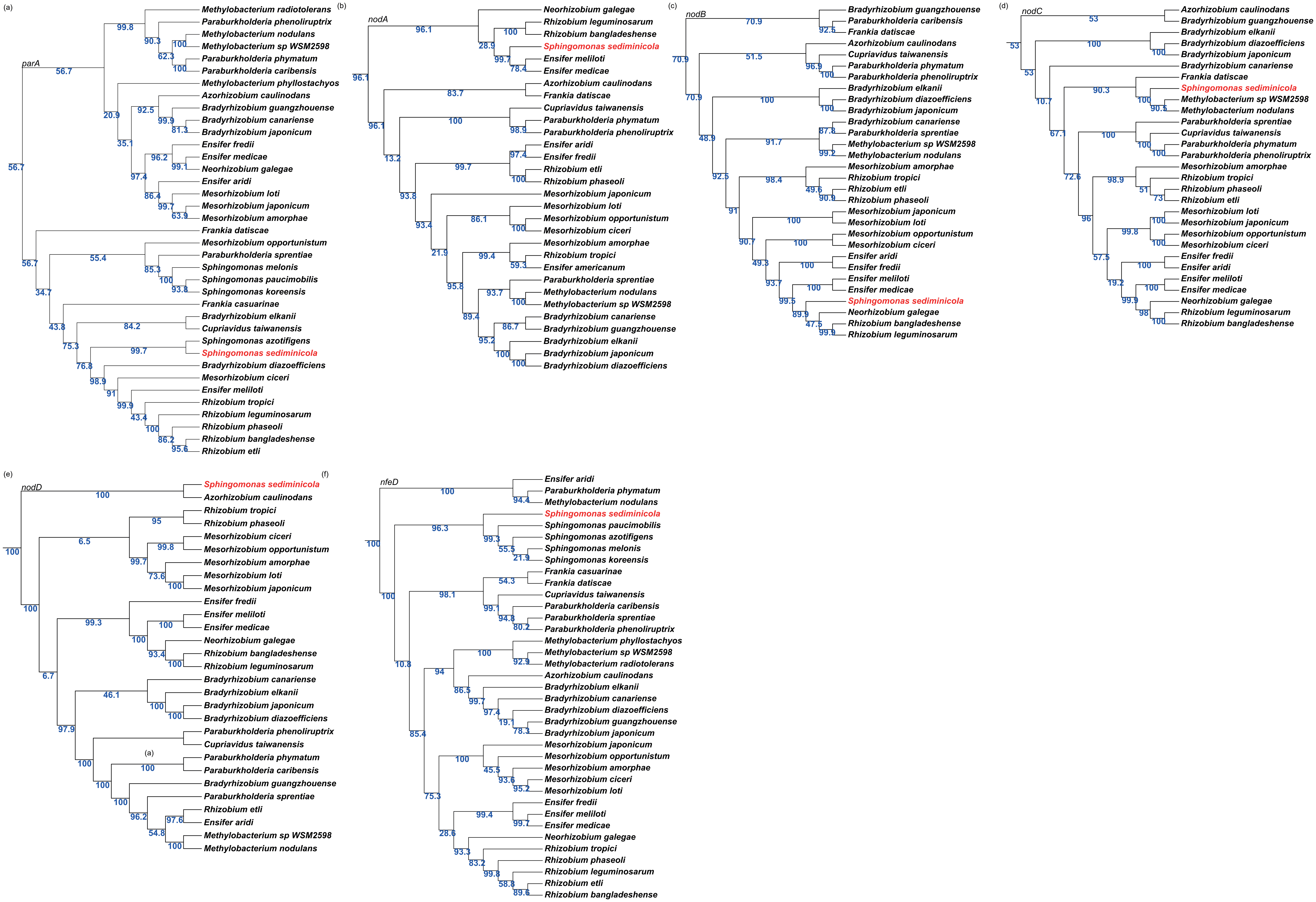

Figure S1. Phylogenetic tree of the multiple sequence alignment of (a) parA, (b) nodA, (c) nodB, (d) nodC, (e) nodD and (f) nfeD sequences from atmospheric N2 fixing symbiotic and non-symbiotic bacterial species. Sphingomonas sediminicola is in red. Bootstrap values of the phylogenetic tree were indicated in blue.
