# Supplementary material for: Sphingomonas sediminicola Is an Endosymbiotic Bacterium Able to Induce the Formation of Root Nodules in Pea (Pisum sativum L.) and to Enhance Plant Biomass Production
Source: Microorganisms. 2023 Jan 12;11(1):199. doi: 10.3390/microorganisms11010199 (PMC9861922; doi:10.3390/microorganisms11010199)
Supplement: Supplementary file 1 [file microorganisms-11-00199-s001.zip › Figure S2.pdf]

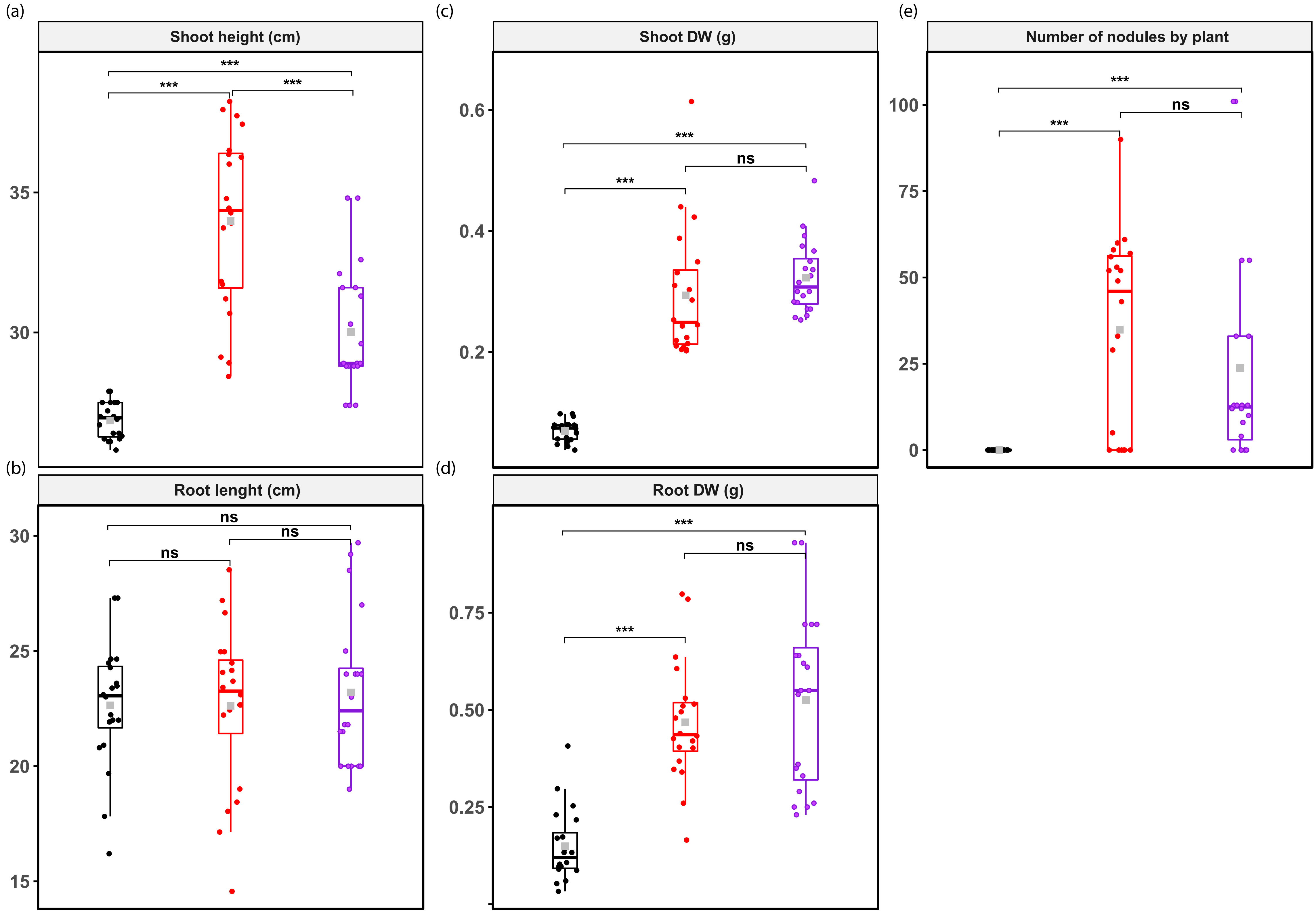

Figure S2: Effect of inoculation with *Sphingomonas sediminicola*Rif [pOPS0385] (purple) or *Rhizobium leguminosarum* (red) on some phenotypic traits of peas compared to uninoculated peas (black) when plants were grown in the absence of nitrogen. (a) Shoot height, (b) root length. (c) Shoot and (d) root dry weight (DW). (e) Number of root nodules per plant. Statistical differences were based on Wilcoxon rank sum tests with Holm's p-adjust. ***, $p < 0.001$; ns, not significant
