# Supplementary material for: Sphingomonas sediminicola Is an Endosymbiotic Bacterium Able to Induce the Formation of Root Nodules in Pea (Pisum sativum L.) and to Enhance Plant Biomass Production
Source: Microorganisms. 2023 Jan 12;11(1):199. doi: 10.3390/microorganisms11010199 (PMC9861922; doi:10.3390/microorganisms11010199)
Supplement: Supplementary file 1 [file microorganisms-11-00199-s001.zip › Figure S3.pdf]

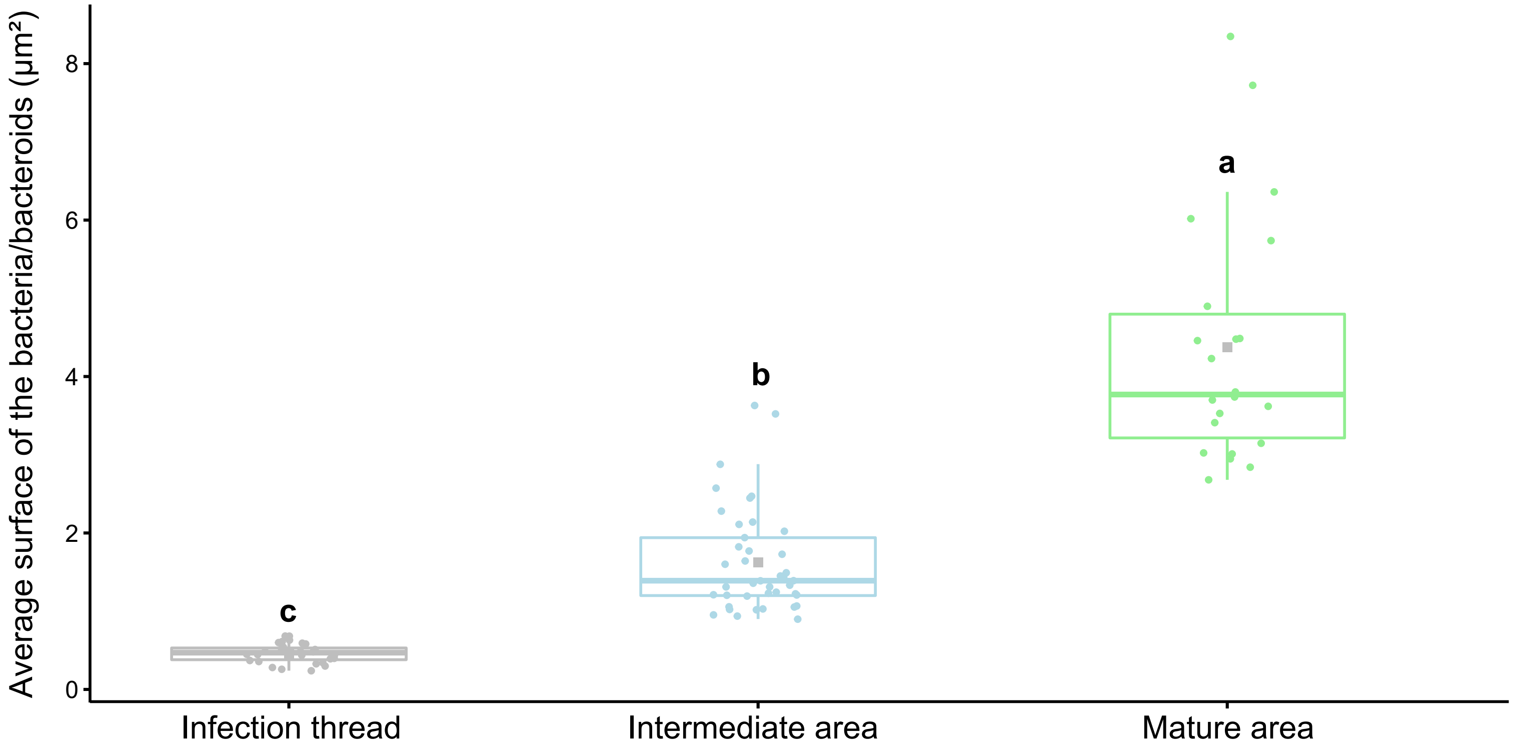

Figure S3. Measurement of the surface area of *S. sediminicola* in three different root nodule zones. Surface areas were measured by TEM image analysis on 30 to 50 longitudinally sectioned bacteria or bacteroids; partial and transverse sections were not included in the measurements. Significant differences (different letters) between infection threads, intermediate and mature zones were determined by ANOVA followed by Tukey-Kramer HSD at a 95% confidence limit.
